# GPS tracking data reveals daily spatio-temporal movement patterns of waterfowl

**DOI:** 10.1186/s40462-019-0146-8

**Published:** 2019-02-25

**Authors:** Fiona McDuie, Michael L. Casazza, Cory T. Overton, Mark P. Herzog, C. Alexander Hartman, Sarah H. Peterson, Cliff L. Feldheim, Joshua T. Ackerman

**Affiliations:** 1 San Jose State University Research Foundation, Moss Landing Marine Laboratories, 8272 Moss Landing Rd, Moss Landing, CA 95039 USA; 2 U.S. Geological Survey, Western Ecological Research Center, Dixon Field Station, 800 Business Park Drive, Suite D, Dixon, CA 95620 USA; 3 California Department of Water Resources, Suisun Marsh Program, 3500 Industrial Blvd, #131, West Sacramento, 95691 CA USA

**Keywords:** Activity budgets, Animal movement, Contagion index, Energetics, Fine-scale movement, High frequency GPS, Movement ecology

## Abstract

**Background:**

Spatio-temporal patterns of movement can characterize relationships between organisms and their surroundings, and address gaps in our understanding of species ecology, activity budgets, bioenergetics, and habitat resource management. Highly mobile waterfowl, which can exploit resources over large spatial extents, are excellent models to understand relationships between movements and resource usage, landscape interactions and specific habitat needs.

**Methods:**

We tracked 3 species of dabbling ducks with GPS-GSM transmitters in 2015–17 to examine fine-scale movement patterns over 24 h periods (30 min interval), dividing movement pathways into temporally continuous segments and spatially contiguous patches. We quantified distances moved, area used and time allocated across the day, using linear and generalized linear mixed models. We investigated behavior through relationships between these variables.

**Results:**

Movements and space-use were small, and varied by species, sex and season. Gadwall (*Mareca strepera*) generally moved least (FFDs: 0.5–0.7 km), but their larger foraging patches resulted from longer within-area movements. Pintails (*Anas acuta*) moved most, were more likely to conduct flights > 300 m, had FFDs of 0.8–1.1 km, used more segments and patches per day that they revisited more frequently, resulting in the longest daily total movements. Females and males differed only during the post-hunt season when females moved more. 23.6% of track segments were short duration (1–2 locations), approximately 1/3 more than would be expected if they occurred randomly, and were more dispersed in the landscape than longer segments. Distance moved in 30 min shortened as segment duration increased, likely reflecting phases of non-movement captured within segments.

**Conclusions:**

Pacific Flyway ducks spend the majority of time using smaller foraging and resting areas than expected or previously reported, implying that foraging areas may be highly localized, and nutrients obtainable from smaller areas. Additionally, movement reductions over time demonstrates behavioral adjustments that represent divergent energetic demands, the detection of which is a key advantage of higher frequency data. Ducks likely use less energy for movement than currently predicted and management, including distribution and configuration of essential habitat, may require reconsideration. Our study illustrates how fine-scale movement data from tracking help understand and inform various other fields of research.

**Electronic supplementary material:**

The online version of this article (10.1186/s40462-019-0146-8) contains supplementary material, which is available to authorized users.

## Background

Animals adjust foraging activity, and therefore movement distances, in relation to available resources [1–3]. Consequently, how they interact with their environments reflects their ecological constraints, habitat or resource requirements and landscape heterogeneity [4]. The scale of animal movements can identify and characterize the environmental or behavioral processes which drive any given movement pattern, as well as represent the energy costs associated with various behaviors or activities. Characterizing movement patterns allows us to better inform and develop theories in related fields of research, such as optimal foraging theory, bioenergetics and estimate time or activity budgets that are directly shaped by movement behavior and distribution of resources [1, 5–7]. The size of, and duration spent in habitat patches reflects the distribution and availability of necessary or used resources for the population in question, a condition that applies across taxa [8, 9]. Therefore, a detailed understanding of how and when animals move about and use the landscape can help develop and improve management and conservation strategies, including habitat distribution and forage quality/quantity needs or objectives.

A variety of intrinsic and extrinsic factors such as phenology, sex, species, resource availability and disturbance affect activity patterns and the need to move. Migratory species need to fatten prior to lengthy migrations and refuel upon return to wintering areas [10–12], while residents have food and habitat needs throughout the year. Hunting activity affects when and how ducks use sanctuaries and refuges, and can limit flight distances ( [13, 14], *but see* [15],), and some species, for example grey teal (*Anas gracilis*) [16] and mallard (*A. platyrhynchos*) [17], demonstrate movement variation according to the distribution of resources.

Waterfowl can rapidly move long distances, which enables access to resources over a large area. Although lengthy waterfowl movements and migrations have been studied in depth (e.g. [18–22]), limitations on the frequency of data acquisition and fine-scale movements have prevented detailed analyses of the relationship between movement patterns and behavior. For example, distances and timing of inter-patch movements varied extensively among studies (see review in [23]), often according to location, year or season and within and between species. Legagneux et al. [24] found mallard forage flight distances (FFDs) in France to vary between 0.5–1.3 km by year, while a study by Bengtsson et al. demonstrated distances of 3–5 km in Sweden. In Louisiana, USA, Cox and Afton [25] found FFDs of northern pintails (*A. acuta*; hereafter pintail; 10.7–18.5 km) that were considerably longer than FFDs of conspecifics in California which Fleskes et al. [26] found ranged from 3.3–7 km. Discrepancies among studies may be caused by divergent methodologies and assumptions associated with limited data from low resolution and low accuracy tracking methodologies (such as radio or VHF), that have been used to carry out much of the prior research. Advances in technology such as high resolution GPS tracking, can provide the level of detail needed to identify and characterize fine-scale space-use by waterfowl and reveal critical habitat. The use of such technology is increasingly common in identifying and characterizing fine-scale movements in animal ecology [27–29]. High frequency movement data are particularly useful for revealing variation in the factors which drive movement [30, 31] and allow us to link movements with behaviors and gain greater insight into species and behavioral ecology [27].

**Fig. 1:**
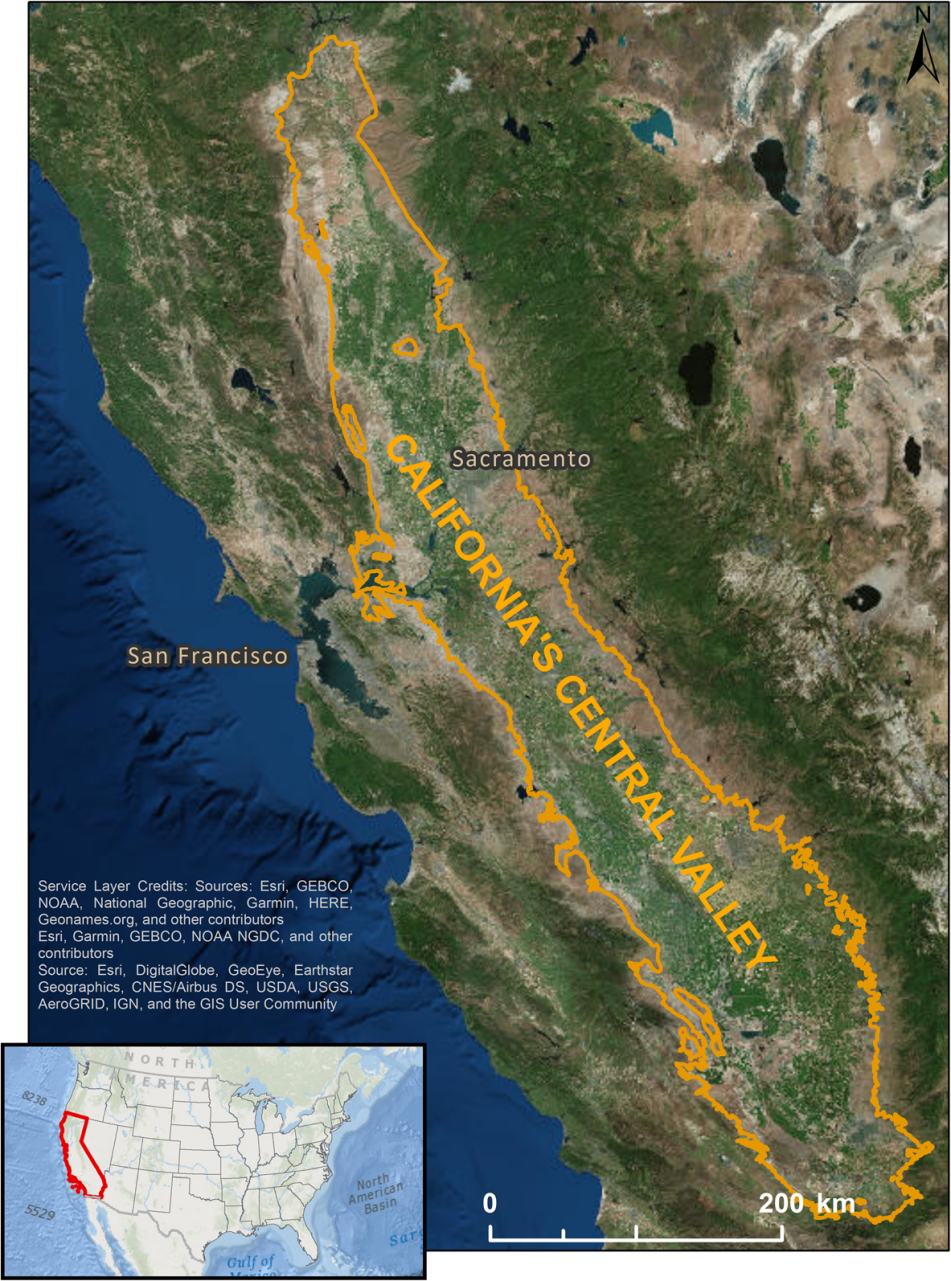
Map showing California’s Central Valley with California outlined on the Continental USA map inset. All 3 species of dabbling ducks (gadwall, mallard and pintail) were tracked with GPS within California producing 2481 ‘bird days’ with complete sets of 48 locations at 30 min intervals which were used to estimate movement and space-use of each species

The broad aim of our study was to characterize fine, spatio-temporal scales of duck movements to identify or inform movement behavior, and determine whether movements varied according to species, sex or time of year. In California’s Central Valley (Fig. 1), the habitat changes considerably throughout the year according to the season, rainfall, agricultural practices and landscape management, which directly influences waterfowl distributions in the large multi-species community [32]. As movement and space-use are expected to diverge as a result of various intrinsic or extrinsic processes such as species ecology and habitat heterogeneity. Therefore, we tracked three species of North American waterfowl (gadwall (*Mareca strepera*), mallard and pintail) with GPS-GSM at the highest frequency possible (30 min interval location data), to precisely quantify daily (‘bird day’- 24 h) movements, space-use and time allocation across 4 seasons (hunt: Nov-Jan; post-hunt (or spring migration): Feb-Apr; summer: May-Aug; and pre-hunt (or fall migration): Sep-Oct). We retained only data collected outside periods when sex or specific life history stages (nesting, brooding, molting and migration) are likely to substantially affect the ability/need to move or fly. We tested hypotheses that species and sexes would differ in these variables. Finally, to determine whether it is possible to use higher frequency data to identify specific bird behaviors, we investigated how time spent in an area was related to locations of, and movement between and within, individual patches.

## Methods

### Study species and area

We focused on tracking the movements of three dabbling duck species within California’s Central Valley: mallard (n female = 38, n male = 7), pintail (n female = 36, n male = 10), gadwall (n female = 18, n male = 0). Mallard and gadwall breed, and are predominantly resident, in California year round, whereas pintail, typically migrate north (March–April) to breed in Canada and Alaska [20, 33–35]. Their return to the Central Valley and Suisun Marsh can vary, but is generally between August and December. We captured ducks in the upland nesting fields on Grizzly Island State Wildlife Area (38.13831°N 121.9781°W), on 8 private duck clubs in Suisun Marsh, and at Howard Slough State Wildlife Area in the Sacramento Valley (39.46726°N, 121.8774° W).

### Field methods and electronic tracking

We began collecting data on January 20th, 2015 and concluded on March 19th, 2017. We captured ducks in Suisun Marsh during 3 time periods: fall, before the hunting season (September to October); winter shortly after the hunting season (February to March); and nesting females in spring/summer (April to July), in order to acquire data throughout their annual life cycle. Additionally, a small number of pintail (*n* = 15) were captured using rocket nets at Howard Slough State Wildlife Area during spring (March to April), just prior to the northward migration. Hunting seasons vary by region but generally begin in mid-October and conclude in late January. Nesting gadwall and mallard females were found on Grizzly Island State Wildlife Area using standard nest dragging techniques [20] and typically captured with large dip nets [36, 37]. We trapped male mallards and pintail on the private duck clubs using baited funnel traps [38] post-hunting season and during the summer (February–March and June–August respectively). Rocket nets were used to capture male and female pintail before the commencement of the hunting season in September–October [36]. Individuals were aged as hatch-year (HY) or after-hatch-year (AHY) based on feather and molt plumage [39] and only adult birds received GPS transmitters**.**

All birds were marked with individually numbered aluminum leg bands and we took morphometric measurements to assess size and weight prior to marking. Transmitter and harness attachments were typically less than 3% of body weight at the time of capture for the lightest marked group (female gadwall) and as low as 1.5% for our heaviest group (male mallards [40]). This ensured that the deployment package weight remained within acceptable body weight limits for birds (3–5%) [40–44]. Captured birds were fit with remotely programmable and solar rechargeable Ecotone® GPS-GSM SAKER L series electronic transmitters that communicate using the cellular (GSM) network. GPS transmitters had a foam pad at the base, weighed 17 g, and measured 58 × 27 × 18 mm. We attached transmitters to adults with back-mounted body harnesses constructed of 5 mm automotive elastic which is less likely to wick moisture to down feathers. Early deployments fasten the elastic ribbon with crimps, later amended to a simple double overhand knot affixed with cyanoacrylic glue, to hold the transmitter in place. Final deployment weight was 18–18.5 g. Total handling time was approximately 20–30 min per bird, and we released each duck at the location of capture.

Transmitters were programmed to take location fixes at 30 min intervals. When the battery reached a minimum critical power level the logger switched to a 6 h interval until it was sufficiently recharged to revert to obtaining locations at shorter intervals. Location coordinates, date, time and battery status were transmitted from the tag to Ecotone (http://telemetry.ecotone.pl) via GSM text message following every fourth location. Because ducks with transmitters may often be in areas outside the range of the cellular GSM network, data were stored on board the transmitter and transmitted when they returned to areas within range of a cell tower. Stored data were backfilled until battery power and GSM signal strength allowed upload.

### Identifying movement and used areas

To ensure the most complete representation of daily movement characteristics possible, we retained only data with a full 24 h period of consecutive position fixes at 30 min intervals. We also omitted the first two weeks of data after marking when movement may be affected by an adjustment period [45–47]. To assess our data according to factors that may influence duck movement and space-use each location was classified with the individual bird identifier (Bird ID), species, sex and which season it occurred in (hunt: Nov-Jan; post-hunt: Feb-Apr; summer: May-Aug and pre-hunt: Sep-Oct). We established day length using sunrise and sunset times classified with the ‘*suncalc’* package in R [48, 49] and categorized all positions as ‘day’ or ‘night’ based upon sunset and sunrise times. Duck movement patterns vary throughout the day [14, 21] with the longest movement distances between forage and roost sites generally expected around sunrise and sunset (dawn and dusk) [21]. As animals are driven by circadian rhythms with movement patterns and behaviors related directly to the rise and set of the sun [14, 50, 51], many previous studies that assess time/activity budgets and finer-scale movement distances do so for 24 h periods [17, 21, 26]. Additionally, waterfowl energetics models, such as ‘SWAMP’, use a daily time step to generate model iterations, and require data that are relevant to that sampling unit – a ‘bird day’ [7]. Therefore, we split movement pathways at sunrise to produce separate 24 h periods (i.e. ‘bird days’). Within any given bird day, the ducks may spend all their time in a single area or they may transition between two or more areas, and we wanted to identify the number and size of these areas, as well as the length of time the birds spent in each. By splitting the ‘bird day’ at the time when the ducks were engaged in conducting longer, forage-roost flights, we increased our likelihood of incorporating all GPS positions that would potentially constitute one of these used areas (for description see next sections). Assessing duck movements using a sunrise to sunrise sampling unit also allowed us to incorporate a common circadian trigger – the start of the photoperiod [52, 53], and to identify locations as ‘*day*’ (sunrise to sunset) or ‘*night*’ (sunset to sunrise), and adjust for seasonal changes in sunrise. It also satisfied our primary objective – to comprehensively evaluate movements within the 24 h ‘bird day’. To assess temporal variation in the occurrence of longer movements, and confirm if they were more likely to occur around sunrise or sunset, we also classified ‘crepuscular’ periods – ‘*dawn*’ - the period from 1½ hours before sunrise to ½ hour after and ‘*dusk*’ – the period beginning ½ before sunset to 1½ hours after; (both encompassing astrological twilight).

We were primarily interested in areas selected and used by the birds and not transit relocations while they were flying. We identified flying behavior through assessment of the speed of the bird between each relocation. When the speed was > 10 km h^− 1^ coming into, and going out of, any given GPS location, it indicated the bird was flying in the moment when the GPS acquired that location (and thus not selecting the habitat over which it was flying), so these were removed. This speed was selected as it is greater than duck walking/swimming (1.8–2.52 kmh^− 1^) speeds [54–56] and lower than flight speeds [57, 58] and was identified using the ‘*adehabitatLT*’ package [59, 60] in R version 3.3.1 [49]. .By identifying flight in this way we could measure the distance traveled from one used habitat to the subsequent used habitat (between segment movements) and include all movements likely to represent foraging or roosting behaviors. We retained for analysis only days that included complete or nearly complete sets of positions at 30 min intervals ([46–49] positions).

In addition, inherently different life history stages present in a duck’s annual cycle can directly influence behavior and movement, and are likely to be sex-specific, so we further filtered our data set to remove those periods when ducks were migrating, molting, nesting and brooding. Migration movements are long directed movements of spatial relocation. In California’s Central Valley, distances of 200 km exceed inter-basin distances and, when achieved across 2 or more hours, and in a generally northerly or southerly direction were identified as migratory movements [19]. Ducks undergo a ‘catastrophic’ molt in which they shed all their primary feathers simultaneously and become flightless for between ~ 25–40 days [61, 62]. As we were primarily interested in analyzing movement when flight is possible, molt periods for all individuals were identified through First Passage Time analyses [49, 63] of telemetry data and removed. We verified nesting behavior, nest success and the beginning of brooding following a successful hatch with weekly nest checks and through visual assessment of hen movements with telemetry data. We concluded cessation of brooding (successfully fledged or failed brood) when a hen flew and remained away for a period of > 2 h, or moved farther/faster than would be possible with ducklings (> 5 km h^− 1^). Data analysis ultimately excluded all movements during migratory, molt, nesting or brooding life history stages.

### Identifying track segments

We used a distance-based path segmentation approach to separate daily relocation paths into spatiotemporally continuous used areas or “segments”. First, we calculated movement distances (m) between all successive GPS fix locations (hereafter called ‘step lengths’). Then, to define ecologically, geographically and behaviorally relevant spatial scales in our data, first we assessed the size of individual management unit areas in state and federal wildlife management areas of California’s Central Valley including Suisun and the Sacramento Wildlife Complex [32]. These areas are often ecologically variable with respect to the plant communities, water depth etc. so we calculated the average radius of those areas and found this to be 295.81 m. Then, we examined the empirical distribution of the natural log of step lengths, using the density function in R [49], following methods by Beatty et al. [64, 65]. Break points in the data were identifiable at 250–300 m for each species. Therefore, we selected 300 m as the distance that a bird would need to move, from the initial location at the start of a segment, to be considered to have switched to a new segment. Thus, all locations for each bird day (sunrise to sunrise) were assigned to one or more temporally and spatially contiguous segments. From this we estimated median step lengths within segments, between segments (from the final location in a segment to the starting location of the subsequent segment), total distance moved within segment and the total distance moved in a bird day. To identify areas and movement patterns during periods predominately associated with feeding activity [66, 67], we evaluated the proportions of segments which occurred entirely during daylight (sunrise to sunset) or nighttime (sunset to sunrise), with the remainder that included both day and night positions designated ‘crepuscular’ segments.

When assessing the distribution of movements throughout the day, the number of segments containing just one or two locations appeared over-represented in our data set. We calculated the expected distribution of the number of locations in a segment given the observed number of segments per day that occurred in our dataset and assuming that a new segment occurred randomly among the 48 locations obtained during the day. We compared this expectation of the number of locations per segment with the actual observed number of locations per segment per day. An excess of short duration segments among our data could occur for two reasons. First, the methodological process of segmenting locations into sunrise-to-sunrise periods may have split longer duration segments into short ones. Alternatively, short duration segments may represent a different, non-random or correlated behavioral process (e.g. disturbance) relative to longer duration segments (e.g., foraging, roosting) that also occurs more frequently than random. We wanted to investigate whether movements into short duration segments represented measurably different spatiotemporal patterns, and therefore behavioral processes, relative to longer duration (> 3 locations) segments. We approached this question using two methods. First, we tested for differences in the movement process by contrasting the average distance that birds moved into short duration segments compared to the distance moved into longer duration segments. Second, we evaluated differences in the spatial arrangement (dispersion) represented by, and dependence, between short duration and long duration segments. Habitat configuration and regional ecology, such as different predator communities, and levels and type of human activity vary across different regions of California. These factors could influence levels of disturbance and consequently, the dispersion of locations used by ducks. Therefore, we evaluated spatial arrangement patterns separately for Suisun, the northern Sacramento Valley, and Southern Oregon North-eastern California (SONEC) regions where data were most extensive.

### Identifying patches

**Fig. 2:**
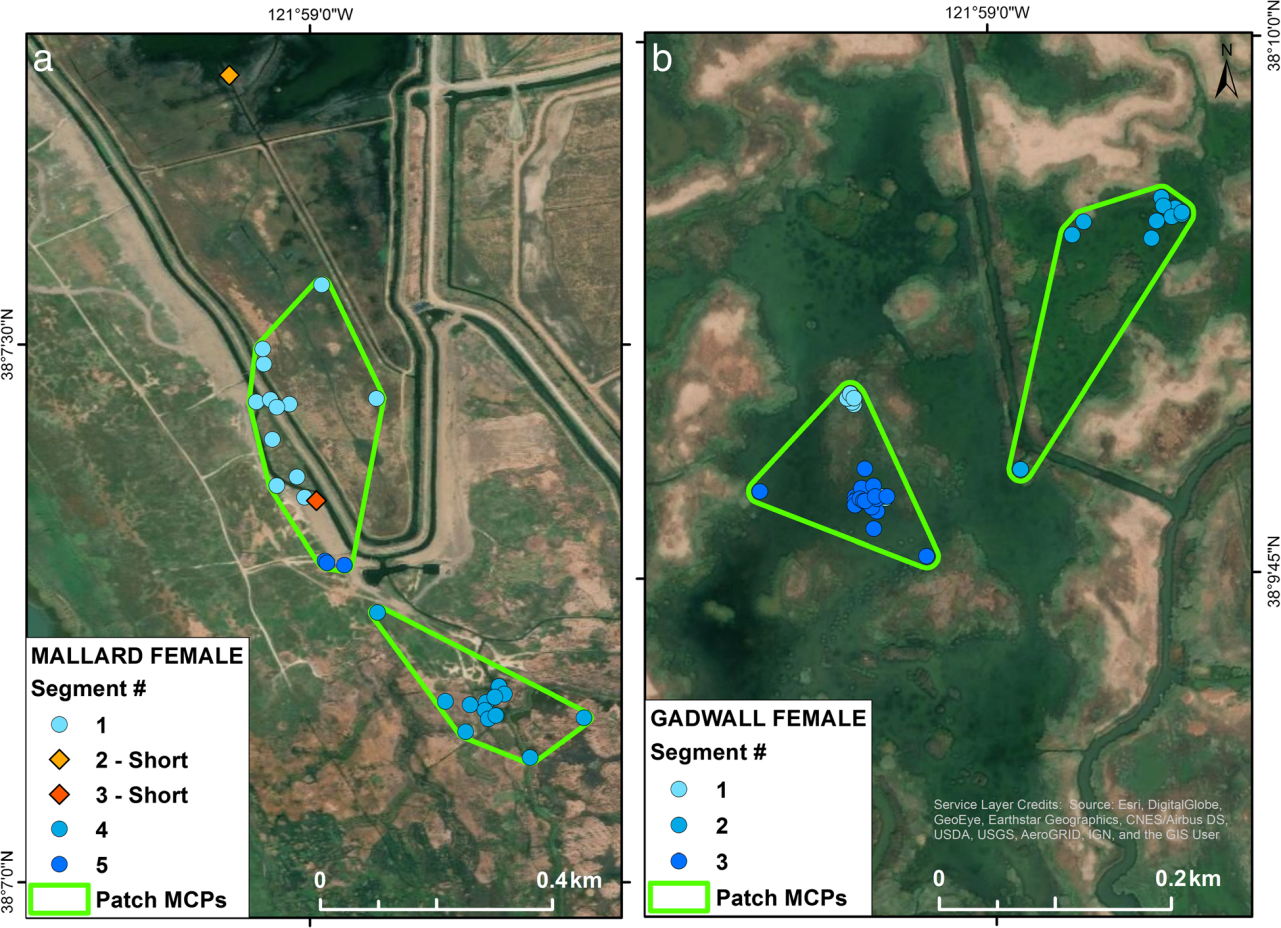
Examples of 30 min GPS positions in a single ‘bird day’ for (**a**) a female mallard and (**b**) a female gadwall, in Suisun Marsh, California. The figure highlights very short movement distances and areas of use, and combination of multiple patches in close proximity. Positions are colored to represent different track segments that are sections of the daily (24 h) track which consist of all positions within 300m of the starting point of that section. Blue circles indicate long duration segments (≥ 3 locations) and orange and red diamonds indicate locations in short duration segments (1 or 2 locations). Colors vary to indicate different segments. Green outlines show minimum convex polygons (MCPs) that combine > 1 segment into a patch, when the birds returned within 300m of the starting location of a prior segment

To understand dabbling duck space use, number and size of individual areas used, total area used and when and how often birds reused the same area within a day, we formed track segments into groups of spatial positions called a ‘patch’ [2], using minimum convex polygons (MCPs). Patches were defined as: 1) all locations occurring within a segment, and 2) a grouping of segments whenever the starting locations for those segments lay within 300 m of each other (for example see Fig. 2). Patches, therefore, are temporally discontinuous whenever 2 or more segments were combined due to proximity (i.e. when a bird returned to an area previously used that day). To evaluate the characteristics of patches we measured the area (ha), number and total size of all patches in a ‘bird day’, the number of relocations (time spent) in the patch, and the proportion of patches occurring in daylight, nighttime or crepuscular periods. Overlap of patch MCPs could occur if locations were intermixed – when any portion of the patch MCP area overlapped with another but starting locations of different segments were greater than 300 m apart. Therefore, in order to correctly estimate the total area used across an entire day, we combined the MCP boundaries around these overlapping patches to avoid totaling the overlapping area twice.

When multiple patches were used within a day, we evaluated whether ducks spent a greater proportion of their time among patches that were closer/more clustered or within more remote/dispersed patches. We developed a ‘spatiotemporal contagion’ index (SCI) to quantify dispersion of time spent among patches by using the ratio of a time-weighted and an unweighted standard distance measure. ‘Standard distance’ quantifies the variance in the spatial location of a set of objects (i.e. the locations in a patch) around their mean center [68, 69], and a time-weighted standard distance biases the mean center toward objects with longer duration of use (i.e. patches with more locations). The ratio of these two measures provides us with an SCI that describes whether a greater proportion of the day was spent among clustered patches (ratio < 1) or among more remotely distributed patches (ratio > 1), similar to the way contagion indices quantify the spatial arrangement of habitat types in landscape ecology [70]. A value of one would indicate that time (i.e. number of locations) is equal among all patches in a bird day or that all patches were equidistant from each other.

### Data processing

Relocation data were collected in the WGS84 geographic coordinate system and re-projected into UTM Zone 10 N projected coordinate system in R version 3.3.1 [49]. Data assessment and manipulation were completed in Microsoft Excel 2013 (Microsoft Corporation, Redmond, WA) and statistical analyses were conducted in R version 3.3.1 [49]. All telemetry locations were buffered by 10 m to account for GPS error. Minimum convex polygons (MCP) were formed using base toolbox and the *XToolsPro* version 16.0 (© XTools Pro, Inc.) extension in ArcGIS for Desktop 10.3.1 [71]. Areal measurements (ha) were calculated for each buffered location or set of locations comprising a patch. As our data were non-normally distributed, all results are presented with the median with 95% confidence intervals (CI) unless otherwise noted.

### Autocorrelation

We evaluated autocorrelation among our step-length data by calculating two separate autocorrelation functions (ACF) using the command ‘acf’ function in R [49]. The variable of interest was step length and individual bird day is the sampling unit. Two ACFs were calculated among all relocations in a given day (sunrise to sunrise period) and the average across all bird days is reported. The two ACFs were calculated from relevant sets of our data: 1) all locations for an individual within a day and, 2) all locations within a single track segment. Since path segmentation is a tool used to identify and separate non-stationary processes [72, 73], it has the natural tendency to reduce autocorrelation relative to that observed in unsegmented non-stationary data where the segments differ in mean value. That does not necessarily imply that segmented tracks are not auto correlated however, only that statistical parameters are constant within each segment, where they are not constant across all relocations. The average Pearson’s coefficient for a first order autoregressive (AR(1)) model across an entire day was 0.208, indicating some, but non-significant (Pearson’s r critical value = +/− 0.28) correlation among successive step lengths. As expected, splitting the tracks reduced autocorrelation among step lengths, resulting in an average AR(1) Pearson’s coefficient within each track segment of − 0.038, therefore, it was not necessary to adjust for over dispersion [74].

### Statistical analyses

To quantify duck movements and variation among the species and sexes for each different season we included only groups with sample sizes of at least 5 individuals and modeled a variety of metrics that estimated distances moved, area used and how time was allocated throughout the day (segments and patches; Table 1). We calculated differences in movement metrics among groups using log-transformed linear mixed models (LMER; ‘lmer’ function in the ‘*lme4*’ package) for most metrics. Discrete data, e.g. counts, were modelled using generalized linear mixed effect models with quasi-Poisson distributions (‘glmmPQL’ function in R package ‘*MASS*’). Data were predominantly obtained from female ducks (84.1%) since summer capture efforts focused on nesting hens so we were only able to model differences between pintail sexes in the hunt and post-hunt seasons and mallard sexes in summer. Since variation in movement patterns among sexes was expected, and male gadwall were not marked for this study, models quantifying differences among species included females only. Statistical comparisons were not made between seasons due to the inability to separate individual random variation from season level variation for some species/sex groups where few individuals occurred across subsequent seasons. The effective degrees of freedom for calculating test-statistics using the Satterthwaite method and significance of predictor variables within generalized linear models was assessed using Wald’s Chi-square (*CAR* package in R [49]) for generalized linear models. Differences between species and sex effects are presented using pairwise comparisons across groups within each season and were adjusted for multiple comparisons using a Tukey adjustment [74]. Where pairwise comparisons among groups were quantified, we estimated the equivalent degrees of freedom for calculating test-statistics using the Satterthwaite method. Results for log-normal and quasi-Poisson distributed variables were back-transformed, where appropriate, allowing interpretation of response variables as median values with asymmetric 95% confidence limit while the differences between group means were back-transformed to allow interpretation as proportional difference between groups [74].

**Table 1:** Median values with upper and lower 95% confidence limits (CI) for models of duck movement of 109 individuals of 3 species of California dabbling ducks. Values are presented by season, for females and males where applicable. Data are estimated from all 30 min interval GPS relocations within 2481 ‘bird days’ (24 h periods)

Model	Season	HUNT (Nov-Jan)				POST-HUNT (Feb-Apr)			SUMMER (May-Aug)			PRE-HUNT (Sep-Oct)		
	Species	Gadwall F	Mallard F	Pintail F	Pintail M	Mallard F	Pintail F	Pintail M	Gadwall F	Mallard F	Mallard M	Gadwall F	Mallard F	Pintail F
	n	7	5	15	6	5	26	6	17	35	6	10	11	18
Total distance per day (km)	Median	4.08	5.65	9.03	9.68	5.85	8.48	5.19	3.35	4.95	4.31	4.00	5.82	5.95
	±95%CI	2.44–6.80	3.15–10.15	7.05–11.56	6.23–15.05	3.68–9.31	6.91–10.41	3.47–7.77	2.67–4.22	4.11–5.94	2.89–6.43	2.85–5.62	4.03–8.39	4.75–7.45
Step length^a^ within segment^b^ (m)	Median	18.99	13.07	16.31	26.46	26.74	26.12	26.46	18.20	19.07	16.72	19.18	14.04	12.16
	±95%CI	13.88–26.00	9.04–18.90	13.48–19.72	20.26–34.57	20.20–35.41	22.90–29.79	20.26–34.57	14.97–22.12	16.47–22.07	11.83–23.64	15.11–24.34	11.08–17.80	10.07–17.80
Step length to new segment (km)	Median	0.73	1.34	1.05	1.12	0.86	0.84	0.96	0.51	0.76	0.71	0.67	1.06	1.13
	±95%CI	0.48–1.09	0.83–2.18	0.87–1.24	0.80–1.57	0.54–1.38	0.69–1.02	0.65–1.46	0.42–0.61	0.66–0.89	0.51–0.98	0.50–0.91	0.78–1.46	0.93–1.38
Segment^b^ duration (hr)	Median	10.29	13.00	8.52	9.16	11.16	7.88	12.59	12.39	10.85	11.98	11.47	11.31	10.01
	±95%CI	7.77–13.62	9.58–17.64	7.55–9.60	7.23–11.59	8.98–13.86	7.15–8.69	10.40–15.24	10.60–14.49	9.74–12.10	9.50–15.10	10.16–12.96	9.66–13.24	9.27–10.81
# segments^b^ per day	Median	4.68	3.73	5.83	5.21	4.33	6.16	3.89	3.99	4.47	4.10	4.12	4.24	4.81
	±95%CI	3.51–6.22	2.64–5.28	5.10–6.68	4.06–6.67	3.42–5.49	5.63–6.74	3.18–4.75	3.45–4.63	4.02–4.96	3.28–5.14	3.63–4.68	3.57–5.04	4.47–5.19
Total area used per day (ha)	Median	3.54	2.23	3.47	4.11	4.46	6.03	4.77	3.36	3.55	3.62	3.93	2.74	2.58
	±95%CI	2.25–5.58	1.33–3.74	2.84–4.25	2.77–6.07	3.28–6.07	5.23–6.97	3.59–6.32	2.75–4.11	3.06–4.13	2.26–5.00	3.10–4.98	2.12–3.55	2.21–3.01
Patch^c^ area (ha)	Median	1.19	1.01	0.89	0.86	1.31	1.37	1.70	1.26	1.03	1.18	1.42	0.77	0.70
	±95%CI	0.80–1.76	0.62–1.64	0.74–1.06	0.61–1.22	0.95–1.22	1.19–1.58	1.25–2.30	1.03–1.55	0.88–1.20	0.85–1.65	1.09–1.84	0.58–1.03	0.59–0.82
# patches^c^ per day	Median	2.77	2.00	2.80	3.27	2.41	3.04	2.14	2.07	2.44	2.36	2.20	2.31	2.56
	±95%CI	2.32–3.31	1.57–2.54	2.59–3.03	2.80–3.82	2.04–2.85	2.87–3.22	1.88–2.44	1.87–2.30	2.27–2.61	2.04–2.72	2.00–2.41	2.03–2.63	2.43–2.70
Overlap (%)	Median	1%	2%	1%	1%	0%	2%	3%	2%	1%	2%	1%	1%	0%
	±95%CI	-1 - 2%	0–4%	0–1%	0–2%	−2 - 2%	0–3%	1–6%	1–3%	1–2%	0–3%	1–2%	0–1%	0–1%
Standard distance (SCI)^d^	Median	−0.02	−0.03	− 0.11	− 0.06	− 0.07	−0.08	− 0.30	−0.28	− 0.11	−0.11	− 0.04	−0.11	− 0.14
	±95%CI	−0.17-0.13	−0.24-0.17	− 0.16- -0.06	−0.19-0.06	− 0.18-0.05	−0.15--0.01	− 0.45--0.16	−0.35- -0.22	− 0.15- -0.08	−0.18- -0.03	− 0.15-0.06	−0.22-0.01	− 0.19--0.08
Revisits (#)	Median	1.15	1.08	1.34	0.82	1.16	1.84	0.86	1.36	1.45	1.19	1.11	1.26	1.13
	±95%CI	0.69–1.90	0.60–1.96	1.06–1.69	0.49–1.35	0.75–1.82	1.59–2.14	0.58–1.28	1.09–1.71	1.23–1.71	0.84–1.69	0.80–1.54	0.87–1.82	0.92–1.39

^a^Step lengths measure the straight-line distance moved in each 30 min interval

^b^Segments are temporally continuous sections of the daily (24 h) track that consist of all 30 min interval positions within 300 m from the starting (anchor) point of that section of the track

^c^Patches are spatially continuous sections of the daily (24 h) track transformed into MCPs to enable area calculation and accounting for revisits to previously used segments

^d^Standard distance measures the dispersion of patches across the landscape when ≥3 patches were used in a 24 h period

To investigate the circadian patterns of duck movement we developed an additional model calculating the probability of longer (> 300 m) movements occurring during dawn, day, dusk or night periods. This model used a logistic generalized linear mixed effects model (GLMER) using Laplace approximation, with a binomial response variable (> 300 m = 1 or < 300 m = 0) and an interaction between categorical time period and group (species or sex) as fixed effect and individual as a random effect using the ‘glmer’ function in the ‘*lme4’* and ‘*contrasts*’ packages in R [49, 75].

Differences in the spatial arrangement of short and long duration segments could indicate different behaviors being displayed or different resources being utilized between the two types of use areas. We evaluated whether short duration segments were randomly distributed with respect to long duration segments using a Monte Carlo bootstrap simulation of one-way nearest neighbor cross-class (NN-CC) distances using the ‘nncross’ function in the R ‘*spatstat*’ package, version 1.51 [76]. We first calculated the average distance from each short duration segment to the nearest long duration segments. Then we randomly assigned locations to each class (short or long duration) while maintaining existing spatial positions and the same proportion of locations in each class. The observed NN-CC distance was compared to distribution of 10,000 bootstrapped replicates to provide a measure of the significance of our data [77]. If the distributions of short and long duration segments are spatially independent the resulting Monte-Carlo *p*-value would be near 0.5. If short duration segments were spatially associated close to, or intermixed with, long duration segments the *p*-value would be near 0.95; and if the short-duration segment distributions were more dispersed and/or in different locations, the *p*-value would be under 0.05. A significantly more dispersed one-way Monte Carlo result could represent either a wider distribution of short duration segments or inclusion of more flying locations as the bird flies between highly clustered long duration segments. However, if the latter situation was occurring we would expect that movement into and movement out of a short duration segment would tend to be unequal, because it is unlikely that most locations would be exactly half way between longer used segments. In addition, movement into a short duration segment should, on average, be shorter than the movement into a longer duration segment if short duration segments represented flight between longer duration segments. Therefore, we tested for equal distances moved into short and long duration segments using an ANOVA in R version 3.4.3 [49].

To determine if movement changed as the time spent within a segment increased we tested the relationship between segment duration (# of locations i.e. time spent, within the segment), and median distance moved between consecutive locations within the segment, using loglinear mixed models with species, sex, season, time of day (day vs. night) and segment duration as fixed effects and a random intercept for each individual. For this analysis, our inference is limited to the interactive relationship between segment duration and whether this effect differed between the day and the night. The statistical difference in slopes estimated by our mixed effect model was tested using a Wald Chi-square test calculated with the Anova function in the ‘*car*’ package in in R version 3.4.3 [49].

## Results

We recorded 118,829 locations over 2481 bird days on 109 individuals of 3 species of waterfowl: pintail (43.7% of days tracked: female = 876; male = 209), mallard (31.7% of days tracked: female = 601; male = 609), gadwall (24.5% of days tracked: female = 186; male = 0), all moving within California (Central Valley Joint Venture Area – 81.0%), SONEC (17.4%) and adjacent areas of California outside the Central Valley (1.6%). Data were separated and analyzed for interspecific (females only) and intersexual differences where possible, by seasons.

### Distance and area used

#### Hunt season

**Fig. 3:**
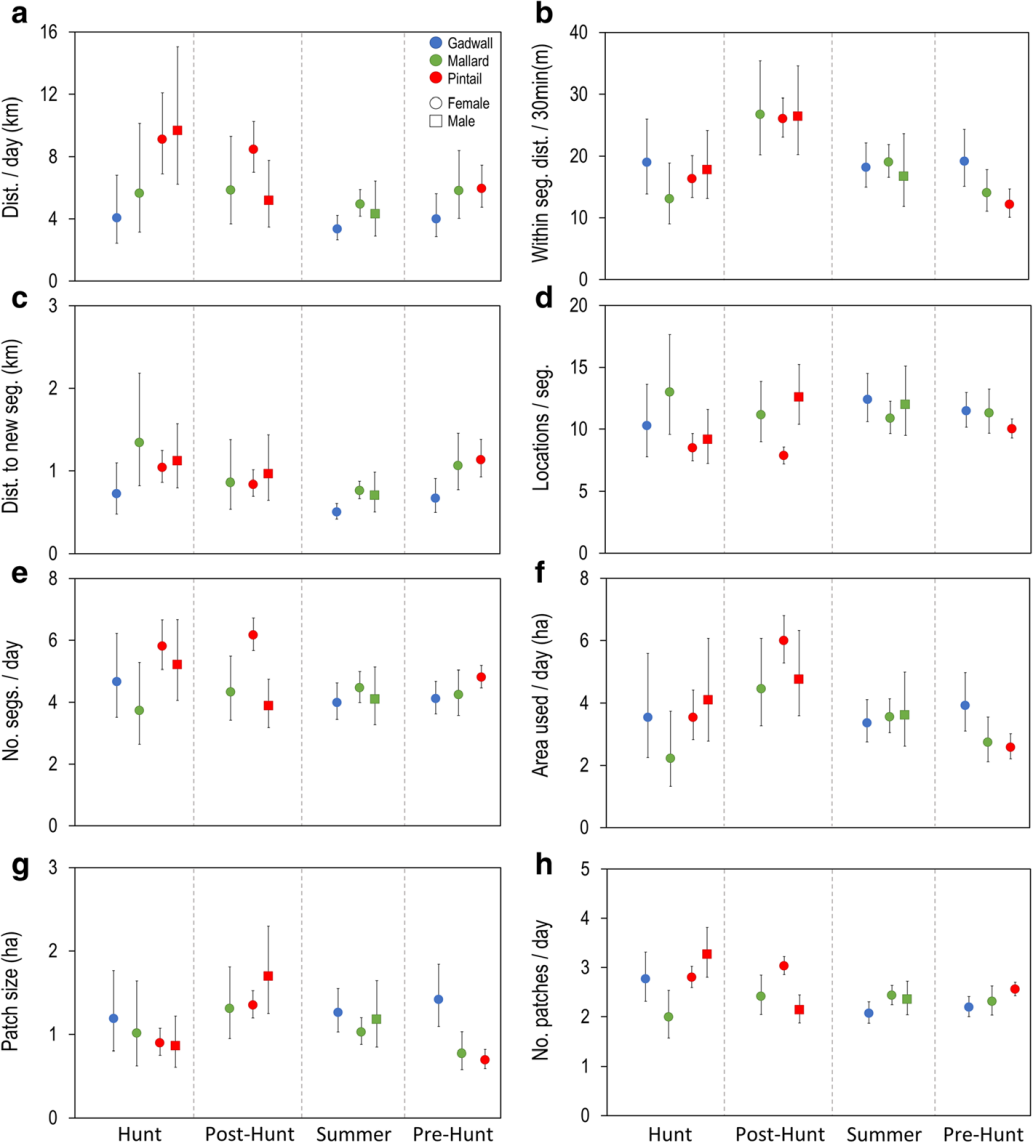
Movement data from GPS tracking of gadwall, mallard and pintail of California’s Central Valley separated by season (hunt: Nov-Jan; posthunt: Feb-Apr; summer: May-Aug; pre-hunt: Sep-Oct) and identified for species (gadwall: blue, mallard: green, pintail: red) and sex (female: circle; male: square) groupings: (**a**) total distance moved per day (24 hr period), (**b**) distances moved in meters per 30 min (step lengths) within track segments, (**c**) distances moved in kilometers per 30 min (step lengths) when switching to a new track segment, (**d**) segment duration (represented by the number of 30 min locations in a track segment; e.g. 10 locations = 5 h), (**e**) number of track segments per day, (**f**) total area used per day in hectares, accounting for revisits and overlap of previously used patches, (**g**) size of individual patches in hectares and, (**h**) number of patches used per day, accounting for revisits and overlap of previously used patches

During the hunt season gadwall moved 45% of the total distance moved by pintail across a bird day (*t*_25.60_ = − 2.812, *p* < 0.05; Table 1, Fig. 3). Mallard spent significantly more time (153%) in segments than pintail (*t*_24_ = 2.651, *p* < 0.05; Table 1, Fig. 3) and used 71% of the number of patches used by pintail (*t*_24_ = − 2.758, *p* < 0.05). There were no intersexual differences in any model.

#### Post-hunt season

During the months of February to April, after the hunting season, mallard spent 142% of the time pintails spent in track segments (*t*_29_ = 3.069, *p* < 0.01; Table 1, Fig. 3) and used 70% of the number of segments used by pintail (*t*_29_ = − 2.878, *p* < 0.01; Table 1, Fig. 3). Mallard also used 80% the number  of patches of > 3 locations, compared with pintail (*t*_29_ = − 2.646, *p* < 0.05; Table 1, Fig. 3).

Pintail sexes differed during this season with females generally moving more than males. This was significant in total distance moved per day, with pintail females moving 163% of the distance that males moved (*t*_19.93_ = 2.264, *p* < 0.05; Table 1, Fig. 3), they spent less time (62%) in segments (*t*_30_ = − 4.452, *p* < 0.001; Table 1, Fig. 3), used 142% the number of segments that males used (*t*_30_ = 4.273, *p* < 0.001; Table 1, Fig. 3), and revisited those segments 214% more frequently (*t*_30_ = 3.683, *p* < 0.001; Table 1, Fig. 3). However, females spent more time among clustered patches than males (SCI; *t*_19.07_ = 3.024, *p* < 0.01; Table 1, Additional file 1: Figure S1).

#### Summer

Differences between non-breeding gadwall and mallard during the summer months of May through August, were limited with gadwall moving less than mallard. Their total distance moved in a day was 67% that which mallards moved and when switching to a new segment (> 300 m movement), this distance was again 67% that moved by mallard (*t*_37.44_ = − 3.624, *p* < 0.001**;** Table 1, Fig. 3). This meant that gadwall spent more time in segments that were more clustered than mallards (*t*_28.93_ = − 4.27, *p* < 0.001**;** Table 1, Additional file 2: Figure S2). In addition, gadwall used 85% of the number of patches that mallards used (*t*_50_ = − 2.435, *p* < 0.05; Table 1, Fig. 3). There were no sex differences in mallards during summer.

#### Pre-hunt season

When moving within a track segment gadwall moved 158% the distance that pintail moved in those 30 min intervals (*t*_29.36_ = 3.054, *p* < 0.05; Table 1, Fig. 3), but when switching to a new segment their FFDs were 59% those moved by pintail. With respect to area used, gadwall used a total area (ha) that was 152% the area used by pintail in a day (*t*_26.07_ = 3.043, *p* < 0.05; Table 1, Fig. 3), used fewer patches (86%) than pintail and those patches were significantly larger (204%; *t*_34.14_ = 3.230, *p* < 0.01; Table 1, Fig. 3). Those patches were also 184% larger than patches occupied by mallard (*t*_22.17_ = 4.796, *p* < 0.001; Table 1, Fig. 3). Finally, in this season we saw the only evidence of significant differences among species or sexes in overlap of patches when gadwall patch overlap was double that of pintail (*t*_356.01_ = 3.615, *p* < 0.01; Table 1, Fig. 3).

### Behaviorally influenced movement

#### Short segments

**Fig. 4:**
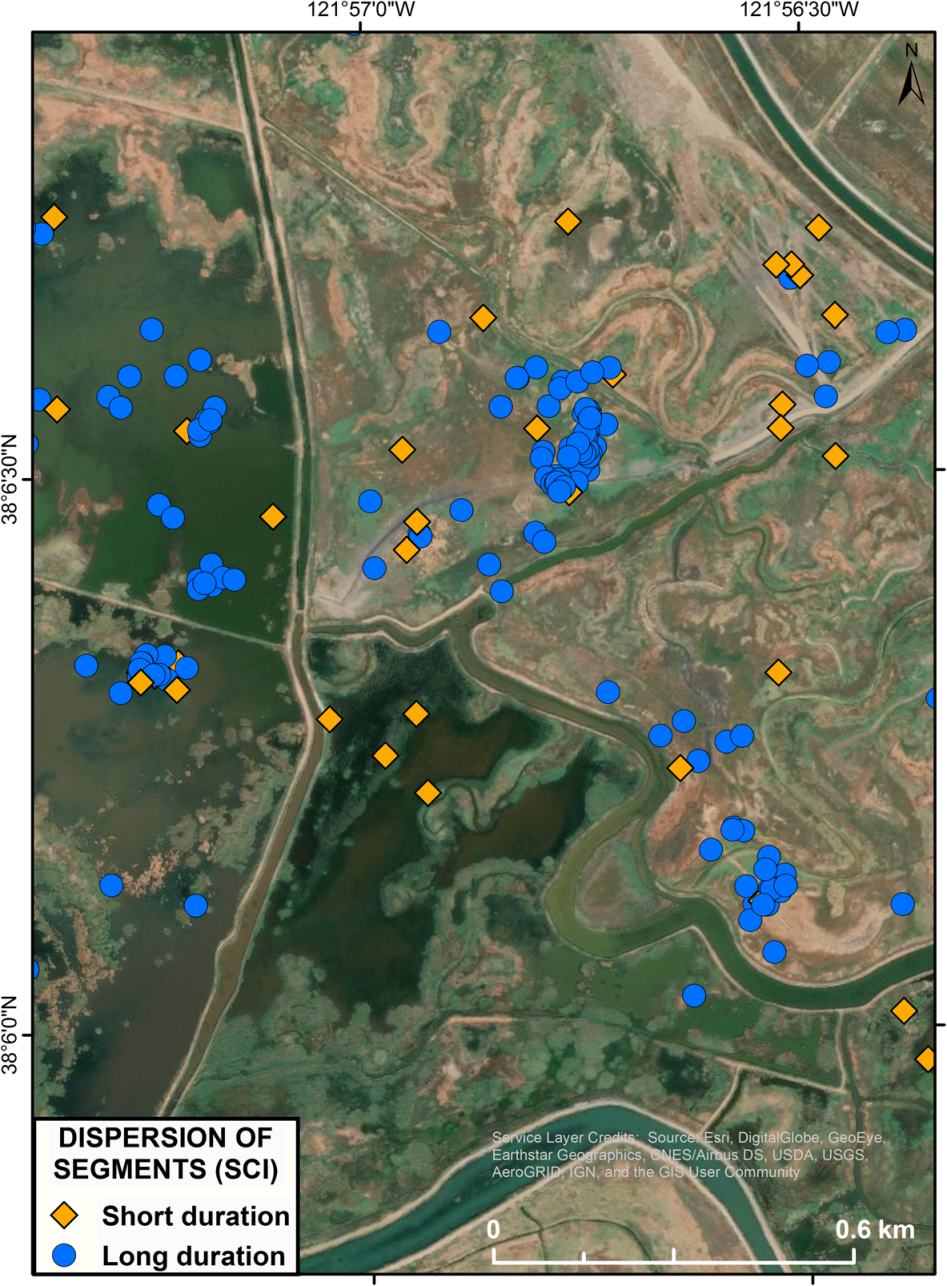
Representation of the spatial dispersion of starting (anchor) points of short (< 1 h; yellow) and long (> 1 h; blue) duration track segments that were used in the Nearest-neighbor cross correlation analysis. Short duration segments were more dispersed across the landscape relative to longer duration segments. Segments are sections of the daily (24 h) track that consist of all positions that are within 300 m from the anchor point of that section of the track

Of the 12,055 track segments, 33% were of short duration (1–2 locations; for example see Fig. 2a). Of these, 9.4% occurred at the beginning or end of the ‘day’, an artefact of our methodological constraint of splitting longer tracks into separate 24 h bird days at sunrise. The remaining portion of short duration segments (23.6%) was substantially higher than would have been expected if the number of locations per segment occurred randomly during the day. Based on parameter constraints from our dataset (i.e. 48 locations/day, and the observed distribution of number of segments/day), only 17.8% of segments would consist of 1–2 locations. Therefore, short duration movements occurred 33% more often than expected. To determine if these short duration segments represented a different kind of movement, we analyzed the distance moved into each segment duration type, which did not differ significantly (LMER: F_1, 9390.19_ = 3.75, *p* = 0.06). We also analyzed the distribution patterns of each and found that the short duration segments were significantly more dispersed than long duration segments according to random bootstrapped Monte Carlo assignments (*p* < 0.0001; Fig. 4).

#### Reduced movement

**Fig. 5:**
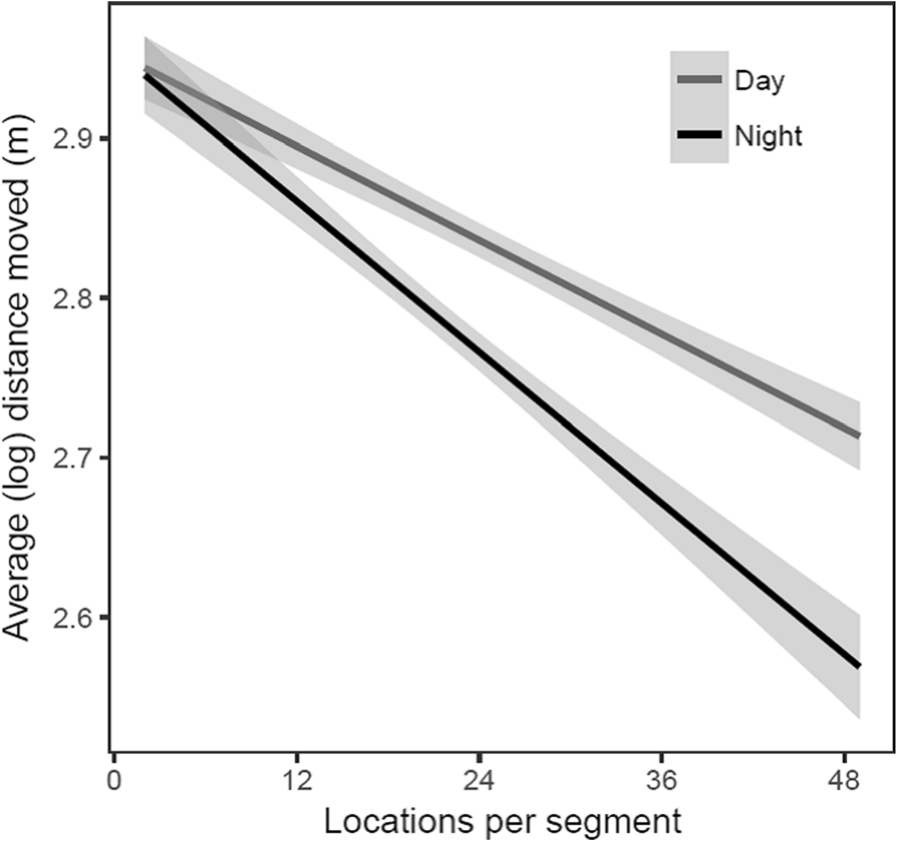
Distance moved between sequential 30 min locations on dabbling ducks within California’s Central Valley. Distance varied according to the amount of time ducks spent in a track segment (2–48 locations), which also differed between day and night. Segments are segments are sections of the daily (24 h) track that consist of all step lengths that are within 300 m from the starting point of that section of the track. As time (i.e. number of locations) in a segment increased, the distance moved in 30 min decreased. This likely indicates an increasing proportion of time during which the birds are stationary (resting/roosting), with a stronger decline at night than during the day

Our high frequency data highlighted a tendency for ducks to reduce movement within segments as the time spent within segments increased. Step length (log) decreased the longer ducks spent in segments both during the day (Fig. 5; GLMER: b = − 0.0052, SE = 0.0004, t106667.1 = − 12.803, *p* < 0.001) and at night (Fig. 5; GLMER: b = − 0.0079, SE = 0.0006, t101970.3 = − 13.1133, *p* < 0.001) when the decline in movement was statistically greater than during the day (Chi-sq = 13.089, df = 1; *p* < 0.001).

#### Time of day movement

**Fig. 6:**
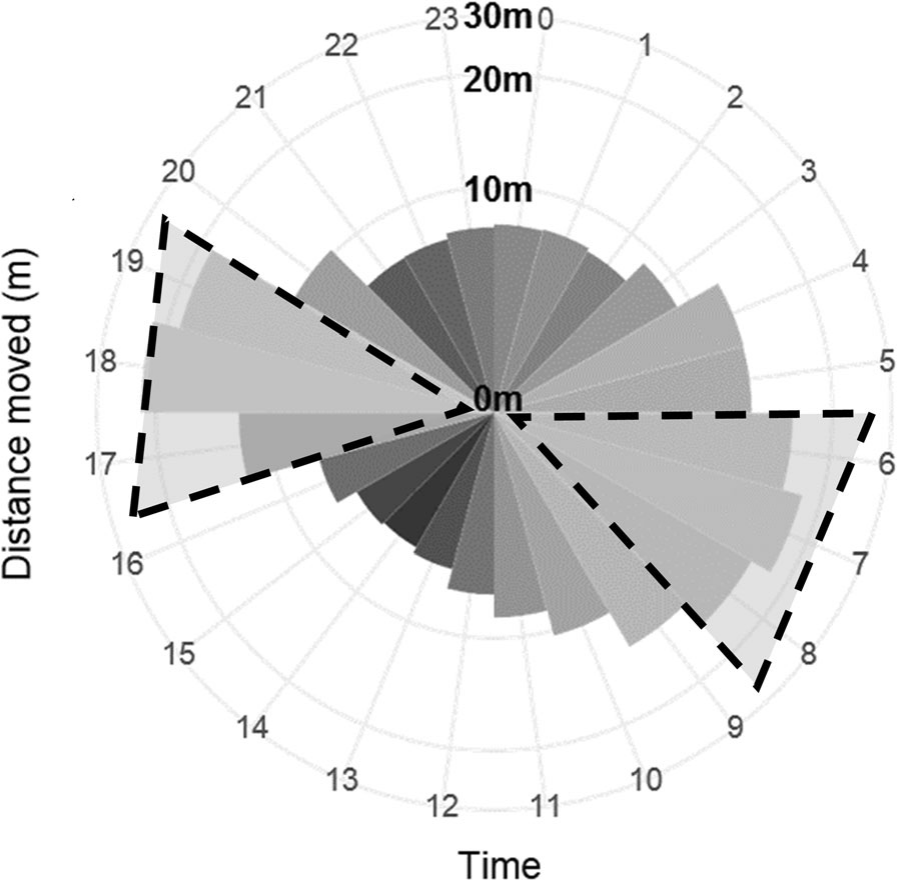
Median movement distances of dabbling ducks of California’s Central Valley obtained with GPS showing 30 min interval movement distances by hour of the day. Data from the entire duration of the study show longer movements were typically made in the hours around dawn (05:30 to 08:30) and dusk (16:30 to 19:30 h; grey shaded triangles with dashed outline). The grey circles represent distance in meters with the inner circle representing 0 m and the outer 30 m. Grey shading varies according to distance

In general, the probability of conducting a foraging flight greater than 300 m was greater for all species at dawn and dusk and least during the night (Fig. 6). In the hunt and post hunt seasons there were no significant differences in the way species responded in different time periods (dawn, day, dusk, night), only overall effects that showed that mallard were 42% less likely to move > 300 m during the hunt season (z = − 2.855, *p* < 0.05) and 56% less likely during the post-hunt (z = − 4.04, *p* < 0.001). During summer and the pre-hunt seasons the species responded differently at different times of day. In summer, gadwall made these longer movements 56% less often than mallard during dawn (z = − 5.424, *p* < 0.001) and dusk (z = − 5.766, *p* < 0.001), and similarly, in the pre-hunt gadwall moved > 300 m 59% less often than pintail during day (z = − 3.528, *p* < 0.05) and 37% less across dusk (z = − 5.273, *p* < 0.001), and 36% less than mallard at dusk only (z = − 3.877, *p* < 0.01). The only evidence of inter-sexual differences was found in the post-hunt season when female pintails were 3 times more likely to move > 300 m than males during the day (z = 7.769.424, *p* < 0.001).

## Discussion

Dabbling ducks in California’s Central Valley moved shorter distances and used smaller areas than expected based on previous tracking studies carried out at lower resolutions or with different methodologies (see review in [23, 26]). Although movement varied by season, daily distances moved, were never more than 10 km (more commonly ~ 3.3–6 km), while inter-patch movements (or FFDs, one of the more commonly measured duck movement parameters) ranged from 0.5–2.2 km across species. Gadwall tended to move less than other species, with FFDs of 0.5–0.7 km, which is shorter than the 2.5 km movements observed in France, in the only other study that estimated FFDs for gadwall [13, 23]. However, when in their patches, gadwall moved more than the other species, resulting in larger spatial areas of use. This is likely related to foraging ecology as gadwall demonstrate divergent habitat selection, foraging patterns and behavior to target different food resources [40, 78, 79]. By contrast, pintail were generally the ‘flightiest’ ducks, using more segments and patches per day, which were revisited more frequently, and demonstrating a higher likelihood of conducting longer flights (> 300 m) than the other species. This resulted in longer per day movement distances and FFDs of 0.8–1.1 km. A study by Fleskes et al. [26], provides the only other estimates for California pintail, and notes longer FFDs of ~ 1.7–7.1 km. The same study estimated mallard FFDs at 2.8–4.8 km which are also longer than those we observed (0.7–1.3 km). These differences may be attributable to habitat variation in the 14 years between the studies, arising from changes in habitat quality associated with enhancement strategies implemented since 1990 [32].

Although, bias from methodological differences in identification of bird locations, is likely to drive dissimilar results, our results also deviate (although to a lesser degree) from the only other GPS studies conducted on dabbling ducks. Mallards in the Netherlands and Sweden were noted to have FFDs of 0.6–2.1 km [17] and < 1–26 km respectively [21]. Inter-regional variation is known to differentially influence duck movement [16, 24] and our estimates of mallard space-use were also considerably less than expected from studies conducted in Europe. Our mallards used a daily area in winter of 2.2 ha (0.02 km^2^) compared with GPS estimated mallard winter daily home ranges of 9.7 ha (0.1–30 km^2^) in the Netherlands [17].

Movement variation by sex was most apparent during the post-hunt season when pintail females moved more than males, used a greater number of used areas that they spent less time in, and revisited more often which made them more clustered. It is the period prior to migration and after the hunting season during which the bulk of the courtship behavior occurs [66]. The pursuit of females by males may cause them to switch ponds more often in the attempt to elude males [50, 80], and this would likely explain these results. We also analyzed an additional movement metric that has not previously been described - the size of the individual patches used throughout a bird day; i.e. each patch used by ducks when not flying - between flighted relocations. The size of these foraging patches was small, approximately 0.7 (pintail females, pre-hunt) – 1.7 ha (pintail males, post-hunt; Table 1) in which ducks spent 5 and 6 h respectively. These infrequent movements, with ducks occupying 3.7 (mallard females, hunt season) - 6.1 (pintail females, post-hunt) track segments per day mean that our data could be augmented with lower frequency data (4–6 locations per 24 h) for those species groups that currently lack high frequency information. This would substantially increase the amount of usable data across the winter months when we more commonly receive fewer data due to reduced day length and hours of sunlight that makes it more difficult for solar panels to recharge batteries.

Patterns and rates of movement influence energy expenditure [81], and this, as well as space-use, is related to the budgeting of time and activity throughout a day. These parameters are employed by energetics models such as the agent-based ‘SWAMP’ [7] and the spatially implicit population based model ‘TRUEMET’ [32]. These models are important management tools that incorporate movement metrics to understand and inform resource needs, with respect to habitat and food requirements, of ducks in the multi-species community of California’s Central Valley. Until now, appropriate data on California ducks was lacking. Consequently, movement and space-use are currently overestimated for these models. Additionally, current time-activity budgets estimate the proportion of the day spent in flight, at approximately 2–6% [50, 51, 80, 82], while our results indicate this to be approximately 0.3% on average. If flight, and the elevated energy expenditure it involves [82, 83], is overestimated, then estimations of energy expended by ducks is likely to be lower than is currently being modeled [82, 84–86]. .Furthermore, these metrics are currently calculated from food habits, body mass and average energy used by a caged bird [87], without empirical estimates of movement. Therefore, the costs of free-living activities, such as various forms of locomotion performed by uncaged birds, have not been included. The models also lack information that directly informs estimates of waterfowl foraging efficiency, habitat and energy requirements [7], including foraging patch selection and patch switching during bouts, while other parameters, such as forager dispersal, were judged qualitatively. Detail from the results of the present study, such as size of areas, duration of use, distances moved, revisits to previously used areas, overlap and clustering of areas, can now supply missing information and quantitatively parameterize and validate these models. We also observed some patterns, such as the relatively common occurrence of revisits to previously used areas (~ 18% of days), and lengthy stays in small areas that demonstrate consistency of resource-use within a day. Currently, variables used in models are for an ‘average duck’, with no allowance for species-specific or behavioral variation. Combined, these results suggest that integrating species-specific data and behavioral components may further improve the accuracy of energetics models.

Duck movements included an abundance of relatively short duration (1–2 locations) movements into new and remote segments (> 300 m) that would not be distinguishable with lower temporal resolution tracking data. These locations could be inflight points inadvertently captured when the bird is transiting between longer duration patches, especially if the short duration segment consists of only a single point. However, if they are followed by a return to a previously used area, or the segment includes two locations, this scenario is unlikely as the distance that must be traveled to reach these short duration segments is beyond the error of our GPS (~ 10 m). Furthermore, while dependent on life history stage, current estimates of time spent flying for ducks is approximately 6% of their day [51]. This time-activity budget seems overestimated given the short movement distances we observed. In either case, the chances of capturing inflight points are relatively small (< 3 positions at 30 min intervals). They may represent a brief foray to investigate alternative foraging sites or a purposeful targeting of those areas for a particular or specialized activity; for example, if these patches represent a habitat that provides a limited and rapidly exhausted resource (Fig. 4). Alternatively, it could reflect temporary relocations to sub-optimal habitat, caused by some kind of disturbance, to which ducks are known to be susceptible, and which causes them to flush from a high quality foraging patch [14, 88, 89]. If this is the case, the spatial and temporal scale of these short segments is considerably less than estimates presented in previous work [90], further underlining the small scale at which these waterfowl interact with the landscape. It is not currently possible to disentangle the trigger of these short duration segments. However, because short flights have increased energetic demands due to the greater cost of take-offs, landings, ascents and descents [83], there are obvious implications for energetics estimations.

Resource selection theory states that when resources are plentiful animals will move less [91–93]. Therefore, where movements are small or infrequent, habitats should be profitable. Movement can be restricted for other reasons such as habitat fragmentation or loss [94, 95], although this can cause movement to increase rather than decrease [96]. However, not only did our ducks demonstrate consistently small movements in general, but they spent more time in clustered areas and reduced movement the longer they spent within an area. These aspects of movement and space-use and surprisingly small-scale habitat exploitation by these ducks suggest that the area of land needed to satisfy this population on a daily basis is considerably smaller than previously thought [26], and that these species spend time in areas where they can reach all necessary resources with relative ease. This implies that either their movements are constrained by habitat limitations and/or that food is not limiting. It is possible that the opposite is true and they are starving and unable or unwilling to move more. However, it is unlikely that food resources in the Central Valley could be so low as to restrict movement. Firstly, agricultural practices of flooding instead of burning rice fields, have increased winter food availability in this system [32]. Secondly, and probably as a result, the body condition of these populations has improved over recent decades. Pacific Flyway ducks are approximately 10% heavier than they were in the 1980’s [97] and have greater average body masses than other North American [98–102] or global [103–107] duck populations. In addition, even though pintail are known to lose mass across the winter months (from November to late January [97, 108]), and mallard and gadwall across the summer breeding season [102, 104], their late season weights (USGS, *unpub. data*) are still largely greater than those of conspecifics in other regions. Therefore, our data provide no clear evidence that food resources are limiting for waterfowl in the Central Valley but rather, that our tracked ducks are able to satisfy their daily energy needs in comparatively small spatial extents.

If food is not limiting, it may be more important for management efforts to focus on developing other limited essential habitats. ‘Waterfowl friendly’ agricultural management programs have increased wetland and food resources [32], while other habitats, particularly “off-season” habitats such as molting wetlands, upland nesting habitat and brood ponds, are not in plentiful supply [20, 26, 109]. The small movements consistently demonstrated by our ducks across seasons and species suggest a mosaic of habitats in close proximity, which meet life history requirements, is critical. Breeding ducks are limited in the distance they can move away from the nest to forage, or when escorting their broods to suitable ponds, and post-breeders are forced to leave the Central Valley to molt [20, 61]. This not only presents a seasonal aspect to management for consideration, but wetland managers could also carefully assess the type, distribution and configuration of available habitat. For example, for the resident breeding species (mallard and gadwall), importance could be placed on increasing availability of currently insufficient nesting and brood habitat, and ensuring brood ponds exist in near enough proximity to the nesting habitat throughout the breeding period. Improving availability of appropriate molting habitat would also benefit the resident species that currently perform molt migrations to SONEC during June to September. Finally, during the hunting season, it may be more beneficial to California’s waterfowl community, to have more small areas of resources than is currently thought, and their proximity to refuges could also be considered [13, 110, 111]. This enormous winter waterfowl population also inhabits the region during fall (Aug-Oct) and spring (Feb-Apr), when a plentiful food supply for post-migration refueling, and pre-migratory fattening are essential [11, 12, 112]. Consequently, natural resource management should reevaluate the juxtaposition of sanctuary, roosting, breeding and feeding areas, minimize inter-habitat distances and, most importantly, emphasize augmenting areas across the landscape to provide critical habitats that are currently lacking.

## Conclusion

Our study’s fine-scale tracking data demonstrate the strikingly small space used by these ducks and provide the most accurate and detailed information obtained to date on these species. That these estimates differ by species compared with those obtained from previous, lower frequency tracking studies, generates revised expectations for life history traits or strategies, movement patterns and behavior and related estimations used in other areas of research. Moreover, by having obtained better information on movement trajectories with higher frequency data, and identified previously undetected movement patterns likely related to specific behaviors, we can begin to develop an understanding of the factors that drive various movements, and how they vary over multiple spatio-temporal scales. Ultimately, accurate assessments of movement are essential to wildlife ecology, helping us to best understand the relationship and interaction between an animal and its environment, the effects of each upon the other [113, 114] and how this influences natural resource management planning and decision-making.

## Additional files

Additional file 1: **Figure S1.** Probability density function and spatial scales based on natural log transformed step lengths for three species of California ducks tracked by GPS between 2015 and 2017. Labels on the x-axis have been back-transformed to display units in meters, following methods by Beatty et al. 2014 and 2015 [64, 65]. The vertical dashed line represents the break in density (300 m) that we used to categorize movements within and between segments. (PNG 29 kb)

Additional file 2: **Figure S2.** Conceptual model of a spatiotemporal contagion index (SCI) used to quantify the distribution of time spent among patches used in day. SCI is based on the ‘standard distance’ metric which quantifies the variance in the spatial location of a set of objects around their mean center. It is calculated by the ratio of the standard distance estimated among all patches without weighting (black dot and dotted circle) and the standard distance calculated when weighting the variance proportional to the amount of time spent in a given patch (grey dot and dotted circle). The dotted circles indicate the variance around the mean centers—the standard distance. When more time is spent in clustered patches (a) the SCI will be < 1; when more time is spent in remotely dispersed patches the SCI will be > 1 and if time is equally distributed the SCI will equal 1. (PNG 162 kb)

## Abbreviations

ACF: Autocorrelation functions
AHY: After-hatch-year
ASY: After second year
CI: Confidence intervals
FFD: Forage flight distance
GLMER: Generalized linear mixed effects model
HY: Hatch-year
LMER: Linear mixed effects model
MCP: Minimum convex polygon
NN-CC: Nearest neighbor cross-class
SCI: Spatiotemporal contagion’ index
SONEC: Southern Oregon North-eastern California
SY: Second year
