# Supplementary figures and images for: GPS tracking data reveals daily spatio-temporal movement patterns of waterfowl
Source: Mov Ecol. 2019 Feb 25;7:6. doi: 10.1186/s40462-019-0146-8 (PMC6388499; doi:10.1186/s40462-019-0146-8)

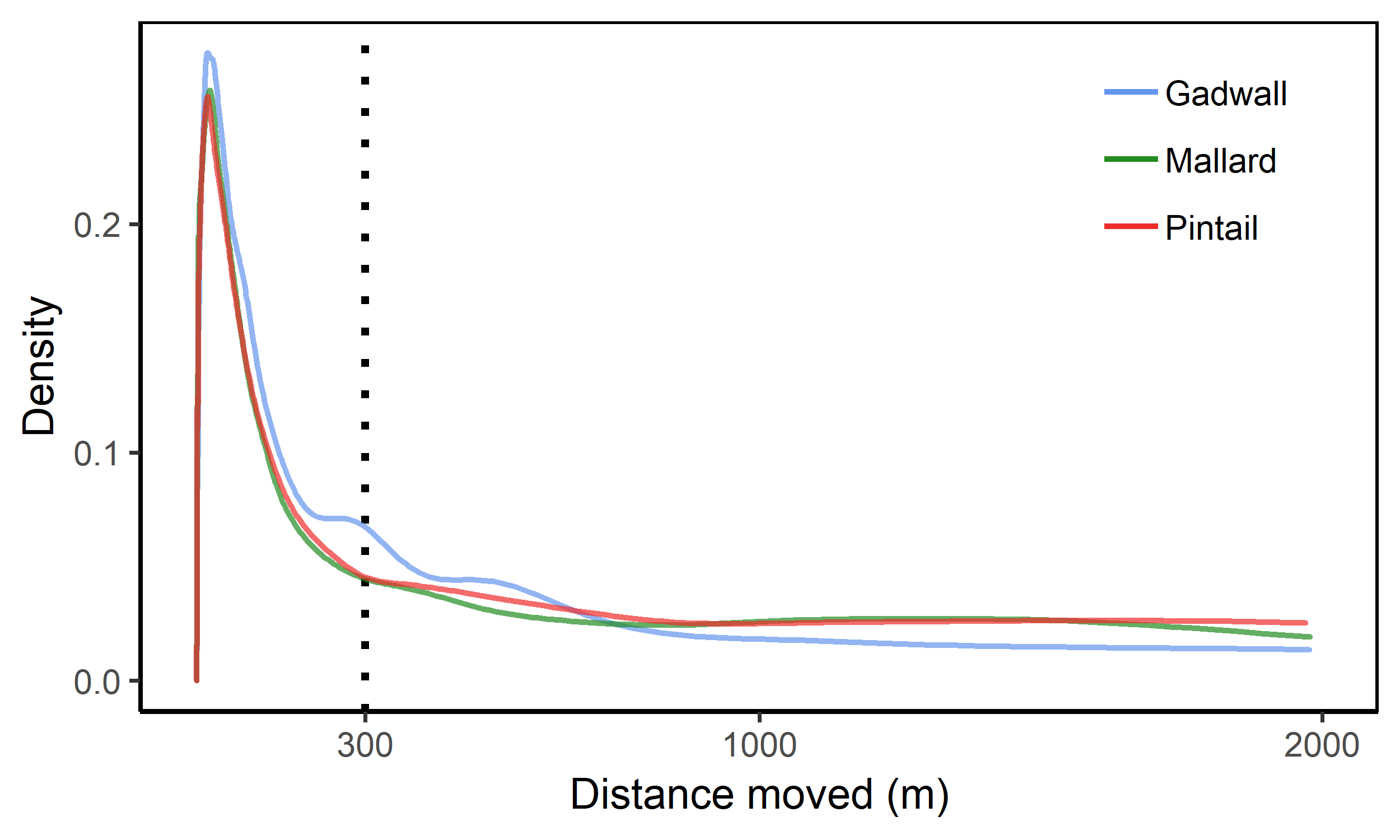

Supplement: Supplementary file 1 — Figure S1. Probability density function and spatial scales based on natural log transformed step lengths for three species of California ducks tracked by GPS between 2015 and 2017. Labels on the x-axis have been back-transformed to display units in meters, following methods by Beatty et al. 2014 and 2015 [64, 65]. The vertical dashed line represents the break in density (300 m) that we used to categorize movements within and between segments. (PNG 29 kb) [file 40462_2019_146_MOESM1_ESM.png]

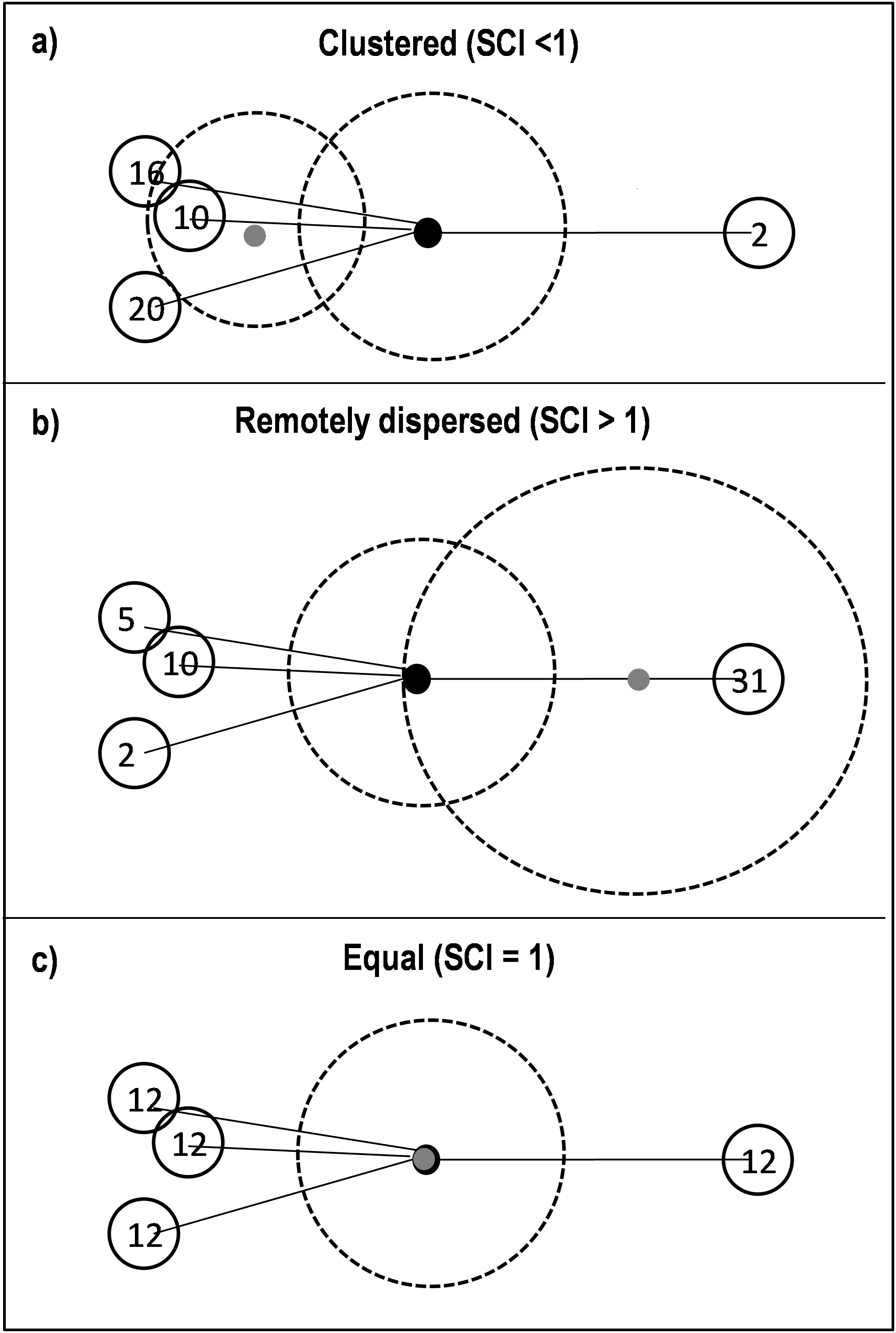

Supplement: Supplementary file 2 — Figure S2. Conceptual model of a spatiotemporal contagion index (SCI) used to quantify the distribution of time spent among patches used in day. SCI is based on the ‘standard distance’ metric which quantifies the variance in the spatial location of a set of objects around their mean center. It is calculated by the ratio of the standard distance estimated among all patches without weighting (black dot and dotted circle) and the standard distance calculated when weighting the variance proportional to the amount of time spent in a given patch (grey dot and dotted circle). The dotted circles indicate the variance around the mean centers—the standard distance. When more time is spent in clustered patches (a) the SCI will be < 1; when more time is spent in remotely dispersed patches the SCI will be > 1 and if time is equally distributed the SCI will equal 1. (PNG 162 kb) [file 40462_2019_146_MOESM2_ESM.png]
